# Tracking motor units longitudinally across experimental sessions with high‐density surface electromyography

**DOI:** 10.1113/JP273662

**Published:** 2017-02-28

**Authors:** E. Martinez‐Valdes, F. Negro, C. M. Laine, D. Falla, F. Mayer, D. Farina

**Affiliations:** ^1^ Department of Sports Medicine and Sports OrthopaedicsUniversity of PotsdamPotsdamGermany; ^2^ Institute of Neurorehabilitation SystemsBernstein Focus Neurotechnology Göttingen (BFNT)Bernstein Centre for Computational Neuroscience (BCCN)University Medical Center GöttingenGeorg‐August UniversityGöttingenGermany; ^3^ Department of Clinical and Experimental SciencesUniversità degli Studi di BresciaBresciaItaly; ^4^ Centre of Precision Rehabilitation for Spinal Pain (CPR Spine)School of SportExercise and Rehabilitation SciencesCollege of Life and Environmental SciencesUniversity of BirminghamBirminghamUK; ^5^ Department of BioengineeringImperial College LondonRoyal School of MinesLondonUK

## Abstract

**Key points:**

- Classic motor unit (MU) recording and analysis methods do not allow the same MUs to be tracked across different experimental sessions, and therefore, there is limited experimental evidence on the adjustments in MU properties following training or during the progression of neuromuscular disorders.
- We propose a new processing method to track the same MUs across experimental sessions (separated by weeks) by using high‐density surface electromyography.
- The application of the proposed method in two experiments showed that individual MUs can be identified reliably in measurements separated by weeks and that changes in properties of the tracked MUs across experimental sessions can be identified with high sensitivity.
- These results indicate that the behaviour and properties of the same MUs can be monitored across multiple testing sessions.
- The proposed method opens new possibilities in the understanding of adjustments in motor unit properties due to training interventions or the progression of pathologies.

**Abstract:**

A new method is proposed for tracking individual motor units (MUs) across multiple experimental sessions on different days. The technique is based on a novel decomposition approach for high‐density surface electromyography and was tested with two experimental studies for reliability and sensitivity. Experiment I (reliability): ten participants performed isometric knee extensions at 10, 30, 50 and 70% of their maximum voluntary contraction (MVC) force in three sessions, each separated by 1 week. Experiment II (sensitivity): seven participants performed 2 weeks of endurance training (cycling) and were tested pre–post intervention during isometric knee extensions at 10 and 30% MVC. The reliability (Experiment I) and sensitivity (Experiment II) of the measured MU properties were compared for the MUs tracked across sessions, with respect to all MUs identified in each session. In Experiment I, on average 38.3% and 40.1% of the identified MUs could be tracked across two sessions (1 and 2 weeks apart), for the vastus medialis and vastus lateralis, respectively. Moreover, the properties of the tracked MUs were more reliable across sessions than those of the full set of identified MUs (intra‐class correlation coefficients ranged between 0.63—0.99 and 0.39–0.95, respectively). In Experiment II, ∼40% of the MUs could be tracked before and after the training intervention and training‐induced changes in MU conduction velocity had an effect size of 2.1 (tracked MUs) and 1.5 (group of all identified motor units). These results show the possibility of monitoring MU properties longitudinally to document the effect of interventions or the progression of neuromuscular disorders.

## Key points

- Classic motor unit (MU) recording and analysis methods do not allow the same MUs to be tracked across different experimental sessions, and therefore, there is limited experimental evidence on the adjustments in MU properties following training or during the progression of neuromuscular disorders.
- We propose a new processing method to track the same MUs across experimental sessions (separated by weeks) by using high‐density surface electromyography.
- The application of the proposed method in two experiments showed that individual MUs can be identified reliably in measurements separated by weeks and that changes in properties of the tracked MUs across experimental sessions can be identified with high sensitivity.
- These results indicate that the behaviour and properties of the same MUs can be monitored across multiple testing sessions.
- The proposed method opens new possibilities in the understanding of adjustments in motor unit properties due to training interventions or the progression of pathologies.

**Abbreviations**
ALS: amyotrophic lateral sclerosis
CCC: cross‐correlation coefficient
CoV_isi_: coefficient of variation for the inter‐spike interval
EMG: surface electromyography
ES: ffect size
HDEMG: high‐density surface electromyography
ICC: intra‐class correlation coefficient
MU: motor unit
MUAP: motor unit action potential
MUNE: motor unit number estimation
MVC: maximum voluntary contraction
SEM: standard error of the measurement
SIL: silhouette
VL: vastus lateralis
VM: vastus medialis
V˙O2 peak : peak oxygen uptake

## Introduction

The neuromuscular system is highly adaptable. Improvements in motor performance can be observed after only a few training sessions (Aagaard, 2003; Selvanayagam *et al*. 2011), while impairments in motor performance due to injury, inactivity or immobilization occur within a few days (Weibull *et al*. 2011). Since short‐term improvements in motor performance are usually not accompanied by changes in muscle structure (Aagaard, 2003), there has been wide interest in studying the neural mechanisms underlying adaptations to training. For instance, the effects of strength and endurance training on motor performance reflect supraspinal and spinal adjustments (Adam & De Luca, 2005; Adkins *et al*. 2006), which influence the neural drive to the muscles, i.e. motor unit behaviour (Vila‐Cha *et al*. 2010).

Investigation of the behaviour and properties of motor units provides a unique insight into the neural code underlying movements (Farina *et al*. 2016). Yet, only a few studies have specifically analysed motor unit adaptations to training (Duchateau *et al*. 2006). This is mainly due to methodological limitations. Classic intramuscular fine wire or concentric needle electromyography only allows recording from few motor units concurrently. Moreover, it is not possible to follow the same motor units across experimental sessions with these classic methods (Carroll *et al*. 2011). Therefore, the sample detected is too small and too variable across sessions to make inferences on adaptations in the motor unit pool of a muscle in longitudinal studies. The problem of a small sample, intrinsic to selective intramuscular recordings, has been addressed recently with novel multi‐channel surface and intramuscular EMG systems that allow for a substantial enlargement of the number of concurrently detected motor units (Muceli *et al*. 2015).

High‐density surface electromyography (HDEMG) systems may also have the potential, not yet exploited, to track motor units across different sessions. This hypothesis is based on the observation that HDEMG provides a spatial sampling of the electrical activity of motor units over the skin plane and the large number of channels allows precise discrimination between different motor units (Farina *et al*. 2008). This spatial ‘signature’ of each motor unit may be used for longitudinal tracking since it can be detected in an almost identical manner once the electrode grid is placed in a similar location over the skin.

The likelihood of this conclusion increases for increasing number of channels since the probability that two motor units detected in different sessions present exactly the same spatial action potentials over tens of channels is negligible. The possibility of tracking motor units longitudinally with HDEMG during voluntary contractions has, however, never been verified.

In this study we aimed to track individual motor units, identified from HDEMG decomposition, across recording sessions performed in different days. For this purpose, we developed a new decomposition technique, as an extension of the convolutive blind source separation approach proposed in Negro *et al*. (2016), with the introduction of the maximization of the cross‐correlation of the motor unit action potential (MUAP) profiles. The approach was specifically designed to detect common sources over multiple sessions. To test the new method, we compared the motor unit action potentials and properties across days as well as pre and post 2 weeks of endurance training. The results revealed, for the first time, the possibility of identifying and studying the same motor units in humans over different days (separated by weeks), which opens new perspectives for studies on the neuromuscular adaptations to training and disease monitoring.

## Methods

### Motor unit identification and tracking

The motor unit identification and tracking method is an extension of the convolutive blind source separation technique recently described in Negro *et al*. (2016), derived from the convolution kernel compensation method (CKC) (Holobar & Zazula, 2007), with a different approach for convergence to the sources (see Negro *et al*. (2016) for further information). Here we adapted the convolutive blind source separation method to extract motor units with multi‐channel action potential shapes maximally similar across sessions.

The convolutive mixture of HDEMG signals can be represented as a linear and instantaneous mixture of the spike trains of the individual motor units and their delayed versions (see Appendix A). Therefore, using an appropriate extension of the matrix of the measurements (multi‐channel EMG signals) and the specific properties of the sources (non‐Gaussianity/sparsity), it is possible to separate the activity of individual motor unit spike trains using techniques of linear instantaneous blind source separation (Negro *et al*. 2016). Briefly, after a de‐correlation/whitening transformation applied to the extended measurements, a fixed point algorithm (Hyvarinen & Oja, 2000) is used to find a projection vector (linear filter) that maximizes the sparsity of the extracted source. The sparsity assumption is well satisfied by the spiking nature of the motor neurons. After a motor neuron spike train is correctly identified, its projection vector is removed from the solution space and the procedure is repeated to extract the next source. Since the measurement matrix is extended, the procedure extracts the original sources and their delayed versions. Therefore, the number and the order of extracted sources are not known *a priori* and depend on the number of iterations, the extension factor, and the spatial characteristics of the EMG signals.

In this study, a new method for the reliable extraction of common motor units in different recording sessions was implemented. After the full blind decomposition was performed on the first recording session, we applied a semi‐blind separation procedure for the remaining sessions, focusing on finding only the sources that had MUAP profiles similar to the ones extracted from session 1. The decomposition procedure converged to the matched motor units first and then extracted motor units which could not be matched across sessions. In this way, it extracted a population of motor units divided in two groups. The first group consisted of the motor units that were tracked across more than one experimental session (tracked motor units); the second group included those units that were identified in only one experimental session (unmatched motor units). The group of unmatched motor units was analysed across sessions with a sample size similar to the one used for the tracked motor units (see Statistics and Results). The normalized cross‐correlation between the MUAP profiles was used as a measure of similarity. For each motor unit identified in session 1, we ran the semi‐blind algorithm on the other sessions until a motor unit with normalized cross‐correlation higher than 0.8 was found. On a limited number of trials (∼15%) multiple matches with a cross correlation > 0.8 were found. In such cases, the algorithm matched the highest cross‐correlated sources and discarded the other matches. Thus, the algorithm maximized the probability of finding the matched motor units across different sessions and considerably reduced the computational load.

In the results presented in this study, we used an extension factor of 16 for the decomposition iteration and 50 samples for computing the similarity measures between de‐whitened projection vectors (original multichannel filters or MUAP profiles). The choice of extension factor was similar to that in Negro *et al*. (2016) for surface EMG signals sampled at 2048 Hz. The number of samples for computing similarity measures (corresponding to ∼25 ms) was chosen to estimate the cross‐correlation using the whole MUAP representation in each channel.

The mathematical details of the approach are provided in Appendix A.

### Experimental tests

Two experiments were designed to test the proposed method and to prove its effectiveness at monitoring changes in motor unit properties compared to the classic approach of averaging results across the full population of identified units in each condition (Vila‐Cha *et al*. 2010; Martinez‐Valdes *et al*. 2016). From now on, the full sample of identified motor units (without matching across sessions) will be referred to as ‘total group of identified motor units’. The first experiment (Experiment I) was designed to prove the reliability of the motor unit properties when estimated over different sessions without interventions on the subjects. This experiment was conducted by measuring motor unit properties over three sessions in 2 weeks. The motor units were tracked by the proposed method and their properties were estimated in each session. The reliability of these estimates was statistically analysed when the motor units were tracked with respect to the total group of identified motor units and also to the unmatched motor units (subset of the total group of motor units that could not be tracked across sessions). The second experiment (Experiment II) was designed to test the sensitivity of motor unit tracking when measures were separated by a training intervention, which could also influence the shapes of the action potentials. Motor unit properties that were expected to change due to training were compared pre and post training, with and without tracking (total group of motor units).

The two experiments provide a strong experimental validation of the proposed method and of its effectiveness.

### Subjects

Ten healthy and physically active men (mean (SD) age: 27 (4) years, height: 180 (8) cm, mass: 78 (10) kg) participated in the first experiment and seven healthy men (age: 28 (2) years, height: 177 (7) cm, mass 78 (9) kg) took part in the second longitudinal experiment (endurance training). All subjects were right leg dominant (determined by asking the subjects which leg they would use to naturally kick a ball). Exclusion criteria included any neuromuscular disorder as well as any current or previous history of knee pain and age < 18 or > 35 years. Participants were asked to avoid any strenuous activity 24 h prior to the measurements. The ethics committee of the Universitaetsmedizin Göttingen approved the first experiment (approval number 24/1/14), performed in Göttingen, and the ethics committee of the Universität Potsdam approved the training intervention (approval number 26/2015), performed in Potsdam, both in accordance with the *Declaration of Helsinki (2004)*. All participants gave written, informed consent.

### Experiment I (repeated measurements)

Participants attended the laboratory on three occasions. Consecutive sessions were 7 days apart and were conducted at the same time of the day for each subject on each occasion. In each experimental session, the participant was seated in an isokinetic dynamometer (Biodex System 3, Biodex Medical Systems Inc., Shirley, NY, USA), with the trunk reclined to 15 deg in an adjustable chair while the hip and distal thigh were secured to the chair. The rotational axis of the dynamometer was aligned with the right lateral femoral epicondyle and the lower leg was secured to the dynamometer lever arm above the lateral malleolus. Maximal and submaximal isometric knee extensions were exerted with the knee flexed to 90 deg. Subjects performed two maximal voluntary contractions (MVC) of knee extension each over a period of 5 s. These trials were separated by 2 min of rest. The highest MVC value was used as a reference for the definition of the submaximal force levels. In each of the three experimental sessions, the submaximal forces were expressed as a percentage of the MVC measured during the same session. Five minutes of rest were provided after the MVC measurement. Then, following a few familiarization trials at low force levels, subjects performed submaximal ramped‐isometric knee extension contractions to 10, 30, 50 and 70% MVC in a randomized order. In each trial, subjects received visual feedback of their knee extension force, which was displayed as a template that had a triangular waveform (e.g. increased isometric leg extension force (ramp‐up) from 0 to 50% MVC in 10 s and decrease of isometric extension force (ramp‐down) from 50% to 0% in 10 s). The contractions at 10% and 70% MVC lasted 14 s (ramp‐up and ramp‐down over 7 s, respectively) while the contractions at 30% and 50% MVC lasted 20 s (ramp‐up and ramp‐down over 10 s, respectively). In this study, we chose to decompose variable‐force contractions, contrary to a previous study where we investigated constant force contractions (Martinez‐Valdes *et al*. 2016). This was done to maximize the impact of tracking units on the reliability of the estimates of motor unit properties. Each force level was performed twice consecutively (with 30 s of rest between repetitions); however, only the second repetition was considered for further analysis. Rest periods of 2, 3, 4 and 5 min were allowed after the 10, 30, 50 and 70% MVC contractions, respectively. One additional MVC was performed at the end of each testing session to evaluate whether the protocol induced fatigue.

### Experiment II (endurance training)

The experimental protocol consisted of a baseline session (i.e. HDEMG recordings, peak oxygen uptake (V˙O2 peak ) determination), a 2‐week intervention of endurance training, and post‐training session. For the baseline testing, prior to training, the subjects performed submaximal isometric knee extensions at 10 and 30% MVC (random order) on an isokinetic dynamometer (CON‐TREX MJ, PHYSIOMED, Regensdorf, Switzerland), following the same procedure presented above (see Experiment I), with the only difference that visual feedback of knee extension force was displayed as a template that had a trapezoidal waveform (5 s ramps with a hold‐phase duration of 20 s). Then, 24 h after the HDEMG‐force measurements, the subjects performed an incremental test to exhaustion on an electronically braked cycle ergometer (Lode Excalibur Sport V2.0, Groningen, the Netherlands) to determine V˙O2 peak  using a gas analysis system (ZAN 600, Nspire Health, Oberthulba, Germany). Following a 3‐min warm‐up at 30 W, the test began with the workload increasing by 6 W every 12 s until volitional exhaustion. The revolutions per minute were maintained between 80 and 90, throughout the incremental test and training sessions. The value used for V˙O2 peak  corresponded to the highest value achieved over a 30 s collection period.

The training protocol commenced approximately 72 h after the incremental test and consisted of six training sessions over 14 days. Each training session was performed on Mondays, Wednesdays, and Fridays. Training consisted of 90–120 min of continuous cycling at 65% of V˙O2 peak  (166.4 (20.1) W). The duration of exercise increased from 90 min during sessions 1 and 2 to 105 min during sessions 3 and 4, and to 120 min during sessions 5 and 6. This protocol has previously been determined to be sufficient to produce an increase in endurance performance and aerobic capacity (Gibala *et al*. 2006). An investigator of the study (E.M.‐V.) supervised all training sessions. The post‐training session (HDEMG recordings and incremental test) was identical to the baseline‐testing procedures and was performed approximately 72 h post training to reduce the effects of post‐training fatigue in all measurements (Gibala *et al*. 2006).

This training regime has been shown to enhance muscle fibre membrane excitability through changes in Na^+^–K^+^ ‐ATPase activity (Green *et al*. 2004). Therefore, we hypothesized that the current protocol would also induce changes in motor unit conduction velocity of the vasti muscles, which have only been previously reported in a longer endurance training intervention (6 weeks) with much lower weekly training volume (Vila‐Cha *et al*. 2010, 2012).

### Data acquisition

Surface EMG signals were recorded in monopolar derivation with a two‐dimensional (2D) adhesive grid (SPES Medica, Salerno, Italy) of 13 × 5 equally spaced electrodes (each of 1 mm diameter, with an inter‐electrode distance of 8 mm), with one electrode absent from the upper right corner. First, the skin of the participants was marked according to guidelines (see Barbero *et al*. (2012) for details), for appropriate electrode orientation. Furthermore, to ensure optimal electrode placement, EMG signals were initially recorded during a brief voluntary contraction during which a linear non‐adhesive electrode array was moved over the skin to detect the location of the innervation zone and tendon regions, as previously described (Masuda *et al*. 1985; Farina *et al*. 2001). After skin preparation (shaving, abrasion and water), the electrode cavities of the grids were filled with conductive paste (SPES Medica, Salerno, Italy) and the grids positioned between the proximal and distal tendons of the vastus lateralis (VL) and vastus medialis (VM) muscles with the electrode columns (comprising 13 electrodes) oriented along the muscle fibres. Reference electrodes were positioned at the right ankle and patella. The location of the electrodes was marked on the skin of the participants using a surgical pen, allowing similar electrode positioning across the experimental sessions.

Force and EMG signals were sampled at 2048 Hz and converted to digital data by a 12‐bit analogue to digital converter (EMG‐USB 2, 256‐channel EMG amplifier, OT Bioelettronica, Torino, Italy, 3 dB, bandwidth 10–500 Hz). EMG signals were amplified by a factor of 2000, 1000, 500 and 500 for the 10, 30, 50 and 70% MVC contractions, respectively. Data were stored on a computer hard disk and offline analysed with Matlab (The Mathworks Inc., Natick, MA, USA). Finally, before decomposition, the 64‐monopolar EMG channels were re‐referenced offline to form 59 bipolar derivations, as the differences between adjacent electrodes in the direction of the muscle fibres.

### Signal analysis

The new method for motor unit identification and maximization of the common sources across sessions described in the first section on Motor unit identification and tracking was applied to extract the MUAPs from the acquired HDEMG data. The discharge times of the identified motor units were converted in binary spike trains in which each data sample was assigned a value of 0 or 1, depending on whether or not the data sample marked the onset of an action potential for a given motor unit. Recruitment and de‐recruitment thresholds for each motor unit were defined as the torque (Nm) at the times when the motor unit began and stopped repetitively discharging action potentials. Discharge times that were separated from the next by > 200 ms were excluded from the estimation of recruitment and de‐recruitment thresholds to avoid aligning the thresholds with noise‐generated discharges (Farina *et al*. 2009). The mean discharge rate was defined as the average discharge rate during the interval of time of activation.

As a quality control, only motor units with a coefficient of variation for the inter‐spike interval (CoV_isi_) < 30% (Laine *et al*. 2015), with a silhouette (SIL) > 0.90 (see Negro *et al*. (2016) for details) were considered for further analysis. SIL is the difference between the within‐ and between‐cluster sums of point‐to‐centroid distances, normalized dividing by the maximum of the two values. SIL is an accuracy index for EMG decomposition similar to the pulse‐to‐noise ratio (see Holobar *et al*. (2014) for details). However, since SIL is a normalized measure, it can be directly associated to the accuracy of the decomposition (Negro *et al*. 2016). Finally, discharges that were separated from the next by < 33.3 ms or > 200 ms (30 and 5 Hz, respectively) were excluded from the mean discharge rate and the coefficient of variation of inter‐spike interval (CoV_isi_) calculations because these discharges are rare for the vasti muscles at submaximal contraction forces and therefore are likely to be due to decomposition errors (Martinez‐Valdes *et al*. 2016).

Motor unit conduction velocity was estimated from double differential derivations of the single motor unit surface multi‐channel action potentials in the longitudinal direction (Farina *et al*. 2001). The channels selected for conduction velocity estimates were based on the criterion of a minimal change in shape of the action potential during propagation. The acceptance criterion for conduction velocity estimates was based on the correlation coefficient of the delayed action potentials (threshold set to 90%). Since the accuracy of motor unit conduction velocity estimates increases with the number of channels used (Farina & Mesin, 2005), we selected the largest amount of channels that showed a cross‐correlation > 90% (3 to 8 double differential channels were used). Additionally, values beyond the physiological range (2–6 m s^−1^) were excluded (Andreassen & Arendt‐Nielsen, 1987). Finally, peak‐to‐peak (p2p) amplitude values were averaged across all the channels of the electrode grid, as presented previously (Martinez‐Valdes *et al*. 2016).

### Statistical analysis

#### General

Results are expressed as mean and standard deviation (SD) unless otherwise stated. Before comparisons, all variables were tested for normality using the Shapiro‐Wilk test. The assumption of sphericity was checked by the Mauchley's test and, if violated, the Greenhouse‐Geisser correction was made to the degrees of freedom. Statistical significance was set at *P* < 0.05.

#### Experiment I

MVCs from the beginning and end of each session were compared using a Student's paired *t* test and the MVCs performed at the beginning of each session were compared by one‐way analysis of variance (ANOVA). Paired *t* tests were used to check the effect of time on the number of tracked motor units (sessions 1—2 *vs*. 1–3 and 2—3 *vs*. 1–3). Therefore, we compared the number of tracked motor units between sessions that were one (sessions 1–2 and 2–3) and 2 weeks apart (sessions 1–3), at each force level (10, 30, 50 and 70% MVC) and muscle (VM and VL), independently.

All motor unit variables (recruitment–de‐recruitment threshold, mean discharge rate and conduction velocity) were analysed for reliability at each force level (10, 30, 50 and 70% MVC) and muscle (VM and VL), independently. The level of reliability of the variables extracted from matched motor units (proposed method), from the total group of identified motor units (independent decompositions using averaged motor unit population samples, including both matched and unmatched motor units), and unmatched motor units (random sample of motor units that could not be tracked across sessions, with a sample size similar to the ones used for tracked motor units) between sessions 1 and 3 was determined by the intra‐class correlation coefficient (ICC _2,1_). ICC scores between 0.8 and 1 were interpreted as ‘excellent’, 0.6–0.8 ‘good’ and < 0.6 ‘poor’ (Bartko, 1966). Additionally, a paired *t* test was performed to detect significant differences between sessions. The absolute reliability was obtained by the standard error of the measurement (SEM = SD√(1 – ICC)). The level of reliability of motor units that were matched across the three sessions was determined by ICC_2,1_, while a one‐way repeated measures ANOVA was used to detect any significant differences between sessions. For the sake of clarity, results are presented only for motor units tracked between sessions 1–3 and 1–2–3. Reliability results (ICC and SEM) were averaged between all force levels (10, 30, 50 and 70% MVC) and presented for each variable and muscle independently.

Finally, the motor unit tracking procedure was also applied across the different force levels within each session. Motor units were tracked between 10 *vs*. 30, 30 *vs*. 50 and 50 *vs*. 70% MVC. The ICC_2,1_ was used to evaluate the reliability of conduction velocity and p2p amplitude values of motor units that were tracked between the different force levels on each session.

#### Experiment II

The estimate of single motor unit conduction velocity was chosen as representative variable to compare pre and post training. The values of this variable estimated for the matched and the total group of identified motor units, pre and post intervention, were compared by paired *t* test. Additionally, the Cohen's *d* was used to estimate the effect size (ES). A Cohen's *d* less than 0.2 was classified as ‘trivial’, 0.2–0.5 as ‘small’, 0.5–0.8 as ‘moderate’, and greater than 0.8 as ‘large’ (Cohen, 1988).

## Results

### Experiment I

Maximal voluntary knee extension force performed at the beginning of each session did not differ between sessions (*P* = 0.099). Furthermore, there was no significant change in MVC across each experimental session (*P* = 0.55, 0.13 and 0.08, for sessions 1, 2 and 3, respectively). The total and average number of accurately decomposed motor units from both muscles (CoV_isi_ < 30% and SIL > 0.9) is presented for each session and force level in Table 1.

**Table 1:** Total of accurately decomposed motor units

		Vastus medialis			Vastus lateralis		
	Force level (% MVC)	Session 1	Session 2	Session 3	Session 1	Session 2	Session 3
Total MUs	10%	50	57	49	66	67	72
	30%	74	83	69	67	75	73
	50%	62	56	59	62	58	59
	70%	31	35	42	23	26	25
Average MU p/subject	10%	5.0 (1.3)	5.7 (2.3)	5.4 (1.5)	7.2 (3.5)	7.4 (3.4)	7.2 (3.0)
	30%	7.4 (2.7)	8.3 (3.1)	6.8 (2.7)	6.7 (3.1)	7.5 (3.9)	7.3 (4.1)
	50%	6.0 (3.1)	5.5 (2.7)	6.3 (3.7)	6.0 (3.7)	5.7 (3.1)	6.2 (3.1)
	70%	3.4 (1.7)	3.6 (2.1)	4.9 (2.9)	3.3 (2.2)	3.3 (2)	3.3 (2.3)

Total and average number of accurately decomposed motor units (MU) (mean (SD)). Results are presented for each muscle (vastus medialis, vastus lateralis), session (1,2 and 3) and force level (10, 30, 50 and 70% of the maximum voluntary contraction (MVC)), independently.

**Figure 1:**
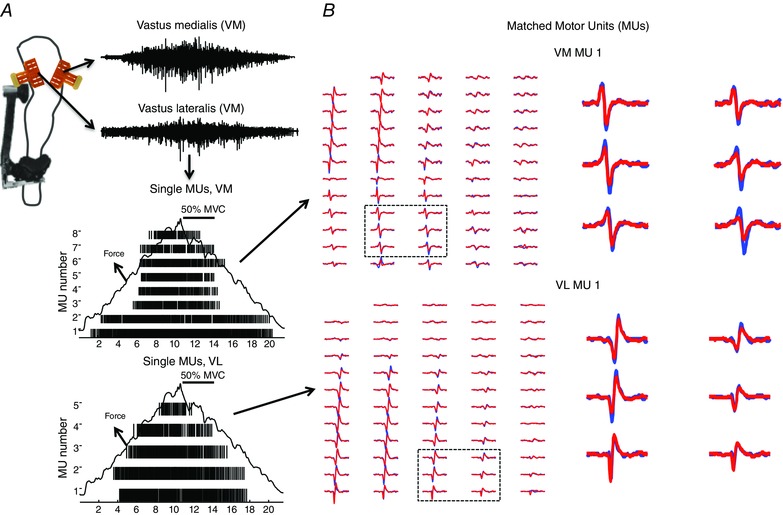
Motor unit decomposition and tracking procedure *A*, high‐density surface EMG signals (64 channels) were recorded from the vastus medialis (VM) and vastus lateralis (VL) muscles during a ramped isometric knee extension (50% of the maximum voluntary contraction (MVC)). The EMG signals were decomposed to reveal the firing activities of single motor units. A schematic representation of the task and motor unit (MU) recording methodology is shown in the left half of the figure. *B*, the procedure developed in the study was then used to identify two matched MUs between the first and the last session of experiment I. The cross‐correlation between the motor unit action potential profiles of the identified MUs was higher than 90%. Multichannel action potentials (59 bipolar channels) of the original (blue) and matched (red) MUs are shown to confirm their similar MU action potential shapes. Two matched MUs are being shown on the right side of the figure (1 for VM, up and 1 for VL, down). For clarity, MU action potentials inside the dashed boxes are zoomed in the right half of the figure. Those matched MUs had cross correlation coefficients > 0.9.

Figure 1 shows an example of the motor unit decomposition and tracking procedure for VM and VL during ramped isometric contractions at 50% of MVC (Fig. 1 *A*). The MUAPs shown in Fig. 1 (which correspond to a motor unit identified in session 1 (blue) and 3 (red)) had a similarity measure (cross‐correlation coefficient) greater than 90% (Fig. 1 *B*), and therefore, they were associated to the same unit. The visual inspection of the action potential shapes confirms the correct automatic identification of the same motor unit. Following the automatic procedure, the number of tracked motor units across two sessions varied between (mean (range)) 21 (6–34) and 23 (6–40), while for three sessions it was possible to track 11 (8–17) and 11 (1–16) motor units for VM and VL, respectively, at each force level (from 10 to 70% MVC), in the 10 subjects (mean number of tracked motor units per subject was 2.2 (0.1) and 1.4 (0.5) for VM, and 2.3 (0.4) and 1.3 (0.1) for VL, across two and three sessions, respectively). Therefore, a mean (range) of 38.3 (16.5–46.5)% and 40.1 (24.5–54.1)% of motor units from those identified by decomposition could be tracked across two sessions, while 21.0 (13.6–25.0)% and 16.3 (4.1–23.4)% could be tracked across the three sessions for VM and VL, respectively. Overall, the number of tracked motor units remained relatively constant at 10, 30 and 50% MVC between all sessions comparisons; however, it decreased at 70% MVC **(**Table 2 **)**, where only 1 motor unit could be tracked across the three sessions for VL. Finally, the number of tracked motor units remained consistent in time since there were no significant differences in the number of tracked motor units between sessions separated by one (1–2 and 2–3) or two weeks (1–3), in both muscles and at all force levels (*P* > 0.05) (Table 2). Further details regarding the total number of matched motor units, the cross correlation coefficients between tracked and unmatched motor units (average cross‐correlation coefficient was calculated from the maximum cross‐correlation coefficient obtained from all possible unmatched motor unit comparisons) and the percentage of tracked motor units from the total across 2 and 3 sessions comparisons are shown in Table 2.

**Table 2:** Number, percentage of tracked motor units and cross correlation coefficients from tracked and unmatched motor units across sessions

		Vastus medialis				Vastus lateralis			
	Force level (% MVC)	Sessions 1‐2	Sessions 2‐3	Sessions 1‐3	Sessions 1,2,3	Sessions 1‐2	Sessions 2‐3	Sessions 1‐3	Sessions 1,2,3
Tracked MU	10%	23 (43%)	22 (42%)	23 (47%)	11 (21%)	22 (33%)	30 (43%)	26 (38%)	16 (23%)
(*N*, %)	30%	34 (45%)	31 (41%)	25 (35%)	17 (23%)	28 (39%)	40 (54%)	31 (44%)	16 (22%)
	50%	19 (32%)	22 (38%)	20 (33%)	8 (14%)	25 (42%)	24 (41%)	16 (26%)	9 (15%)
	70%	15 (46%)	16 (42%)	9 (17%)	9 (25%)	6 (25%)	15 (58%)	9 (38%)	1 (4%)
CCC tracked (%)	10%	88.3 (3.9)	87.4 (3.2)	83.2 (3.1)	87.9 (2.6)	84.8 (3.7)	86 (4.1)	84.4 (6)	86 (2.4)
	30%	84.8 (3.8)	84.4 (4.6)	83.3 (3.3)	86.8 (3.4)	86.2 (4.6)	86.4 (3)	81 (3.7)	87.4 (3.6)
	50%	84.2 (3.5)	83.9 (4.5)	83.6 (5.8)	85.1 (3.7)	85.1 (4.9)	86.9 (3.3)	81 (3.9)	85.4 (4.5)
	70%	83.2 (4.2)	85.6 (2.5)	81 (3.9)	85.6 (1.4)	83.3 (4.2)	85.6 (2.5)	81 (3.9)	80
CCC unmatched	10%	58.7 (4.7)	59.3 (3.7)	59.9 (4.6)	59.3 (4.2)	53.6 (5.7)	55.1 (4.2)	55.9 (4.8)	54.9 (4.7)
(%)	30%	65.6 (6.7)	64.5 (7.3)	64.4 (6.9)	64.8 (6.7)	59.6 (6.1)	59.4 (5.2)	57.2 (5.5)	58.7 (4.3)
	50%	68.5 (2.6)	68.4 (2.8)	68.2 (3.9)	68.9 (2.5)	62.3 (5.2)	66.5 (4.8)	62.8 (6.5)	63.9 (3.7)
	70%	68.6 (4.4)	68.7 (4.9)	63.7 (7.9)	67.1 (4.3)	63.6 (8.4)	66.9 (8.3)	62.6 (7.4)	63.9 (7.4)

Total number (*N*) and percentage of tracked motor units (MUs). Cross correlation coefficients (CCC) (mean (SD)) are presented for each session comparisons at each force level for matched and unmatched motor units (sample of units that could not be tracked across sessions). The number of tracked MUs (%) represents the percentage of MUs that could be tracked from the total number of accurately identified MUs between sessions. Percentages of tracked MUs from sessions 1,2,3 were obtained by averaging the total number of decomposed MUs across the 3 sessions (Table 1). Note that (SD) for vastus lateralis at 70% MVC (Sessions 1,2,3) is not shown, as only 1 MU could be matched across the 3 sessions.

**Figure 2:**
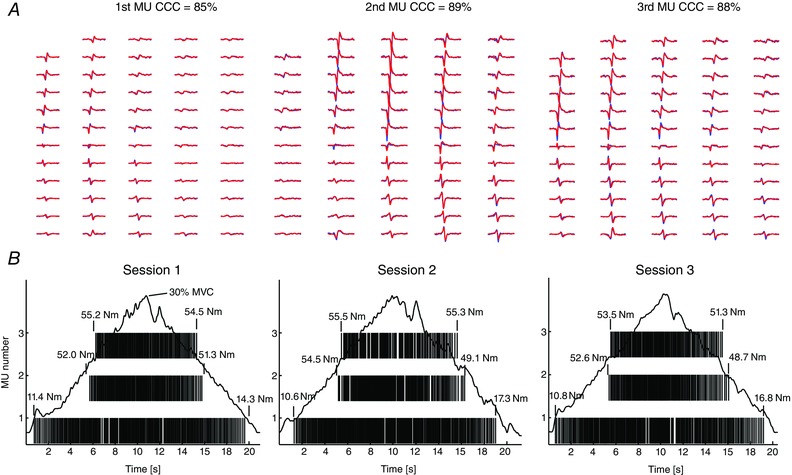
Motor unit tracking across sessions *A*, multichannel surface action potentials of 3 different vastus medialis motor units (MUs) that were tracked across the three sessions. The cross correlation coefficients (CCCs) of the MU action potential profiles between the three sessions can be seen above. For the sake of clarity MU action potential matching is presented between two sessions only. MU action potentials extracted from the first session are presented in blue while matched action potentials from the second session are presented in red. *B*, discharge times of each matched MU during ramped contractions at 30% MVC during the 3 sessions, note the similarity of their recruitment and de‐recruitment thresholds.

The absolute values of the variables extracted from the motor units that were matched between the three sessions are presented in Table 3. Overall, mean discharge rates and conduction velocity increased with force and presented values within physiological ranges, while the recruitment thresholds were similar to the de‐recruitment thresholds (Table 3). A representative example of MUAPs corresponding to three different VM motor units (identified from session 1) that could be tracked across the three sessions with a high similarity measure (cross‐correlation coefficients > 80%) is shown in Fig. 2 *A*. The discharge timings of each matched motor unit, with their corresponding recruitment and de‐recruitment thresholds (expressed as Nm torque) for each session are shown in Fig. 2 *B*. Across sessions, the estimates of recruitment and de‐recruitment thresholds for these matched motor units were stable, as expected. These results were confirmed by the good to excellent levels of reliability (ICCs > 0.60) found for the recruitment–de‐recruitment thresholds, mean discharge rate and conduction velocity of all the tracked motor units in both muscles and across all force levels (see Tables 3 and 4). These results were consistent when variables were compared between two **(**session 1 *vs*. 3, Table 4) or three sessions (sessions 1,2,3, Table 5). These reliability indexes were substantially greater than those computed from the total group of identified motor units and from the unmatched motor units (see Tables 4 and 5), strongly supporting (together with the shape similarity over all channels) the matching performed by the proposed method. None of the variables (from matched, total and unmatched motor units) changed significantly across sessions (*P* > 0.05).

**Table 3:** Motor unit variables in absolute values

		Vastus medialis			Vastus lateralis		
	Force (% MVC)	Session 1	Session 2	Session 3	Session 1	Session 2	Session 3
Recruitment threshold (Nm)	10%	14.8 (3.9)	14.9 (5.4)	14.2 (5.6)	9.5 (5.2)	9.8 (5.4)	10.3 (5.6)
	30%	32.0 (15.2)	32.6 (14.4)	32.6 (13.0)	23.6 (16.9)	23.9 (15.0)	24.1 (16.4)
	50%	71.6 (33.3)	72.7 (36.3)	69.5 (29.1)	70.0 (29.5)	71.6 (34.8)	70.2 (28.2)
	70%	105.0 (22.9)	104.7 (30.8)	110.3 (33.6)	77.1	81.1	78.7
De‐recruitment threshold (Nm)	10%	11.2 (4.9)	12.0 (3.9)	11.4 (4.5)	9.0 (4.2)	9.0 (4.8)	10.0 (4.1)
	30%	35.4 (12.4)	37.6 (12.3)	37.3 (10.4)	24.9 (15.6)	25.2 (16.3)	25.1 (14.1)
	50%	75.9 (33.2)	76.4 (27.5)	75.5 (36.5)	73.9 (27.1)	79.6 (29.8)	76.9 (32.2)
	70%	117.9 (32.6)	120.7 (37.3)	120.7 (39.8)	113.0	110.7	115.6
Mean discharge rate (Hz)	10%	9.4 (1.3)	9.3 (1.3)	9.2 (1.4)	9.7 (1.6)	9.7 (1.8)	9.6 (1.7)
	30%	10.5 (1.0)	10.7 (1.1)	10.3 (0.7)	10.6 (1.1)	10.7 (1.2)	10.7 (1.1)
	50%	12.0 (2.3)	12.0 (2.3)	11.9 (2.0)	10.8 (1.4)	11.2 (1.9)	11 (1.6)
	70%	15.0 (3.1)	14.9 (2.8)	14.7 (2.1)	11.1	11.7	11.6
Conduction velocity (m s^−1^)	10%	4.4 (0.4)	4.4 (0.4)	4.3 (0.3)	4.2 (0.3)	4.2 (0.3)	4.3 (0.3)
	30%	4.5 (0.2)	4.5 (0.2)	4.5 (0.3)	4.3 (0.2)	4.4 (0.3)	4.4 (0.2)
	50%	4.8 (0.6)	4.8 (0.5)	4.7 (0.3)	4.7 (0.4)	4.7 (0.4)	4.7 (0.4)
	70%	4.9 (0.5)	4.9 (0.4)	4.7 (0.4)	4.3	4.4	4.4

Motor unit (MU) variables results (mean (SD)) for MUs matched between sessions 1‐2‐3. Results are presented for each muscle (vastus medialis, vastus lateralis) and force level (10, 30, 50 and 70% of the maximum voluntary contraction (MVC)), independently. Note that (SD) for vastus lateralis variables at 70% MVC is not shown, as only 1 MU could be matched across the 3 sessions.

**Table 4:** Reliability of tracked, total and unmatched motor units from sessions 1 and 3

	Matched MUs				Total MUs				Unmatched MUs			
	Vastus medialis		Vastus lateralis		Vastus medialis		Vastus lateralis		Vastus medialis		Vastus lateralis	
	ICC	SEM	ICC	SEM	ICC	SEM	ICC	SEM	ICC	SEM	ICC	SEM
Recruitment threshold (Nm)	0.92 (0.89–0.96)	5.1 (1.8–8.5)	0.92 (0.88–0.94)	4.5 (1.5–6.3)	0.75 (0.54–0.95)	5.8 (1.6–10.2)	0.63 (0.41–0.92)	7.0 (1.5–11.5)	0.29 (0.15–0.73)	14 (4.2–21.3)	0.44 (0.34–0.49)	14.5 (3.4–22.7)
De‐recruitment threshold (Nm)	0.86 (0.71–0.95)	6.3 (2.4–8.8)	0.87 (0.82–0.92)	6.6 (2.0–12.4)	0.57 (0.43–0.70)	10.1 (2.2–14.4)	0.66 (0.52–0.93)	7.1 (2.7–13.7)	0.36 (0.21–0.50)	16.3 (4.2–24.1)	0.46 (0.41–0.60)	15.3 (4.7–24.8)
Mean discharge rate (Hz)	0.77 (0.72–0.83)	0.8 (0.6–0.9)	0.87 (0.78–0.91)	0.6 (0.6–0.7)	0.56 (0.39–0.73)	1.1 (0.8–1.4)	0.61 (0.42–0.89)	1.0 (0.7–1.5)	0.31 (−0.11–0.59)	1.6 (1.1–2.3)	0.38 (0.21–0.54)	1.5 (1.0–2.5)
Conduction velocity (m s^−1^)	0.84 (0.83–0.87)	0.18 (0.16–0.21)	0.88 (0.84–0.99)	0.12 (0.07–0.12)	0.66 (0.43–0.86)	0.21 (0.16–0.25)	0.56 (0.51–0.67)	0.22 (0.14–0.27)	0.25 (0.05–0.45)	0.35 (0.3–0.39)	0.30 (0.15–0.57)	0.64 (0.44–0.95)

Reliability values are averaged across all contraction levels (10, 30, 50 and 70% of the maximum voluntary contraction) and presented as mean (range) for each variable and muscle (vastus medialis, vastus lateralis). Between sessions comparisons were non‐ statistically significant for all variables at all force levels, for both muscles (*P* > 0.05). ICC, intra‐class correlation coefficient; SEM, standard error of the measurement.

**Table 5:** Reliability of tracked, total and unmatched motor units from all sessions

	Matched MUs				Total MUs				Unmatched MUs			
	Vastus medialis		Vastus lateralis		Vastus medialis		Vastus lateralis		Vastus medialis		Vastus lateralis	
	ICC	SEM	ICC	SEM	ICC	SEM	ICC	SEM	ICC	SEM	ICC	SEM
Recruitment Threshold (Nm)	0.92 (0.89–0.97)	5.2 (1.7–9.6)	0.93 (0.91–0.96)	4.8 (1.4–9.7)	0.81 (0.73–0.94)	6.4 (1.9–9.6)	0.73 (0.65–0.74)	6.2 (2.0–9.7)	0.29 (0.16–0.39)	14.5 (5.2–19.8)	0.42 (0.13–0.66)	14.6 (3.6–22.4)
De‐recruitment Threshold (Nm)	0.81 (0.75–0.92)	7.1 (2.2–17.6)	0.89 (0.83–0.93)	4.8 (1.7–8.4)	0.70 (0.67–0.82)	9.3 (3.9–14.4)	0.73 (0.62–0.88)	7.2 (1.6–13.5)	0.38 (0.27–0.49)	16.2 (3.9–25.8)	0.47 (0.27–0.59)	15.5 (3.8–25.2)
Mean discharge rate (Hz)	0.83 (0.63–0.94)	0.6 (0.6–0.7)	0.84 (0.74–0.90)	0.6 (0.5–0.6)	0.70 (0.58–0.82)	0.9 (0.6–1.1)	0.76 (0.64–0.87)	0.8 (0.5–1.1)	0.30 (0.07–0.59)	1.5 (1.1–2.4)	0.48 (0.26–0.62)	1.4 (0.8–2.0)
Conduction Velocity (m/s)	0.83 (0.78–0.87)	0.16 (0.12–0.21)	0.88 (0.83–0.94)	0.10 (0.10–0.11)	0.73 (0.61– 0.85)	0.32 (0.17–0.21)	0.66 (0.57–0.77)	0.2 (0.13–0.28)	0.16 (−0.29‐0.36)	0.4 (0.35–0.58)	0.39 (0.32–0.46)	0.44 (0.35–0.56)

Reliability values are averaged across all contraction levels (10, 30, 50 and 70% of the maximum voluntary contraction) and presented as mean (range) for each variable and muscle (vastus medialis, vastus lateralis). Between sessions comparisons were non‐statistically significant at all force force levels, for both muscles (*P* > 0.05). ICC, intra‐class correlation coefficient; SEM, standard error of the measurement. Note that reliability for VL at 70% MVC was not calculated (for matched motor units results), since only one motor unit could be tracked across the three sessions.

Finally, for VM and VL, an average of 14 (3) motor units could be tracked between the different force levels within each session (10 *vs*. 30, 30 *vs*. 50 and 50 *vs*. 70% MVC). This represented 24 (6)% of the motor units identified between those force levels. As expected, the tracked motor units showed high cross correlation coefficients (average 91.1 (1.1)%) and good to excellent levels (ICCs > 0.60) of reliability for conduction velocity and p2p amplitude (Table 6).

**Table 6:** Number, percentage and reliability of tracked motor units across the different force levels within a session

	Vastus medialis				Vastus lateralis			
Force levels MVC%	Motor Units (*N*, %)	CCC (%)	CV ICC	p2p amp. ICC	Motor Units (*N*, %)	CCC (%)	CV ICC	p2p amp. ICC
10 *vs*. 30	13 (21%)	90.7 (0.3)	0.88 (0.81–0.93)	0.82 (0.78–0.84)	12 (17%)	93.2 (0.7)	0.94 (0.89–0.95)	0.82 (0.69–0.93)
30 *vs*. 50	13 (19%)	90.2 (0.8)	0.72 (0.60–0.95)	0.73 0(.59–0.84)	19 (29%)	91.4 (0.1)	0.88 (0.80–0.92)	0.93 (0.91–0.94)
50 *vs*. 70	15 (31%)	90.0 (0.4)	0.91 (0.88–0.94)	0.77 (0.64–0.96)	11 (26%)	91.0 (0.1)	0.92 (0.83–0.97)	0.87 (0.74–0.96)

Total number (*N*) and percentage (extracted from the total number of motor units identified between force levels) of tracked motor units across the different force levels (10 *vs*. 30, 30 *vs*. 50 and 50 *vs*. 70% MVC) within each session. The cross correlation coefficients (CCC) (mean (SD)) and intra‐class correlation coefficients (mean (range)) for conduction velocity (CV) and peak‐to‐peak (p2p) amplitude are also presented. For sake of clarity, results are averaged across all sessions.

### Endurance training

After the intervention, incremental cycling peak power output significantly increased from 347.4 (63.2) W to 370.3 (56.9) W, *P* = 0.0004, ES = 2.6. V˙O2 peak  also increased significantly after intervention from 45.1 (6.7) ml kg^−1^ min^−1^ to 48.4 (4.6) ml kg^−1^ min^−1^, *P* = 0.031, ES = 1.1. Peak torque did not differ pre and post intervention (pre: 249.4 (71.6) Nm *vs*. post: 245.7 (59.6) Nm, *P* = 0.5008, ES = 0.3).

**Figure 3:**
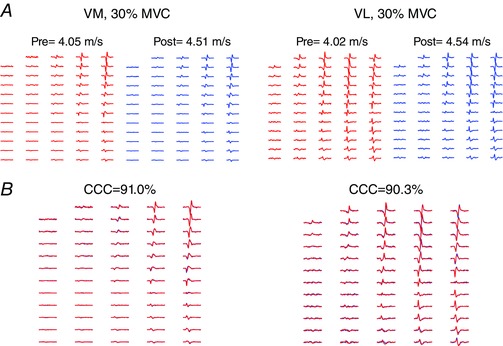
Motor unit tracking and changes in conduction velocity *A*, vastus medialis (VM) and vastus lateralis (VL) motor unit action potentials (MUAPs) that were identified by the tracking algorithm before (pre, red) and after (post, blue) the endurance intervention at 30% of the maximum voluntary contraction (MVC) force. Conduction velocity values can be seen above the MUAPs. *B*, cross‐correlation of the VM and VL MUAPs identified pre and post training. Cross‐correlation coefficients (CCCs) from tracked motor units can be seen above the matched MUAPs. Note the similarity in action potential shape for the tracked motor units despite the large increases in conduction velocity.

For VM, a total of 57 and 77 motor units could be decomposed (CoV_isi_ < 30% and SIL > 0.9), while for VL a total of 59 and 52 units were decomposed at 10% and 30% MVC, respectively. From these units, 44.1% and 41.4% could be tracked post‐training for VM and, 66.7% and 42.5% could be tracked for VL at 10% and 30% MVC, respectively (average cross‐correlation coefficient of 87.0%). Figure 3 shows the motor unit tracking procedure from a representative subject at 30% MVC pre and post intervention. Even though both VM (Fig. 3 *A*) and VL (Fig. 3 *B*) showed a large increase in conduction velocity (10.2% and 11.5% increase, respectively), the shape of their MUAPs remained consistent between pre‐ and post‐testing sessions as confirmed by the large cross correlation coefficients between MUAPs (91.0% and 90.3% for VM and VL, respectively).

**Figure 4:**
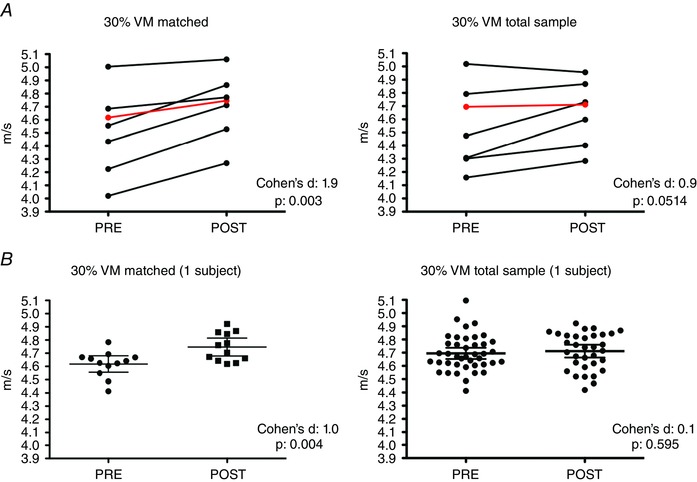
Changes in conduction velocity for tracked and total group of identified motor units *A*, motor unit conduction velocity (CV) values from the vastus medialis (VM) at 30% of the maximum voluntary contraction (MVC) from *n* = 7 subjects, previously (PRE) and after (POST) an endurance training intervention. Left graph shows results obtained with tracked motor units, while right graph shows the results obtained using the total group of identified motor units (CV values were averaged per subject and compared PRE and POST intervention). The effect size and *P* values of the two procedures are shown in the lower right corner of all graphs. The red line depicts an example of one subject that showed an increase in CV of matched motor units (left), which is masked when using the total sample of identified motor units (right). *B*, matched (left) and total sample of identified (right) motor units (mean and 95% confidence interval), from the same subject depicted in *A* (red line). The 12 matched motor units from this subject show a clear intervention effect (left graph), which is not possible to distinguish when using all decomposed motor units (CV values are extracted from all the motor units decomposed pre and post intervention (two repetitions per session)).

Finally, conduction velocity was compared pre–post training to check for the sensitivity of the proposed motor unit tracking method to changes induced by training. For VM, motor unit conduction velocity increased significantly with training when computed for the matched motor units at both 10% (pre: 4.19 (0.27) *vs*. post: 4.37 (0.28) m s^−1^, *P* = 0.013, ES = 1.3) and 30% MVC (pre: 4.51 (0.32) *vs*. post: 4.71 (0.25 m s^−1^, *P* = 0.003, ES = 1.9). These differences were smaller for the total group of identified motor units at both 10% (pre: 4.22 (0.28) *vs*. post: 4.31 (0.22) m s^−1^, *P* = 0.0585, ES = 0.9) and 30% MVC (pre: 4.54 (0.31) *vs*. post: 4.65 (0.24) m s^−1^, *P* = 0.0514, ES = 0.9), for which significant differences were not found. To explain the difference in the results for the matched and total group of identified motor units, Fig. 4 shows individual motor unit conduction velocity results (pre and post training) of the seven participants when using matched (Fig. 4 *A*, left) and total group of identified units (Fig. 4 *A*, right) at 30% MVC (VM). The data from all subjects presented in Fig. 4 *A* (left) show a clear intervention effect when tracking the same motor units that was masked when the motor units were not matched (Fig. 4 *A*, right), with two subjects showing no effect of the intervention without tracking. One of these subjects is highlighted in red (Fig. 4 *A* and *B*). The results for the highlighted subject can be seen in Fig. 4 *B*. The twelve matched motor units (Fig. 4 *B*, left) of this subject showed a clear intervention effect with a large effect size (*P* = 0.004, ES = 1.0). However, this difference could no longer be observed when using all motor units (*P* = 0.595 (unpaired *t* test), ES = 0.1, Fig. 3 *B*, right). Similarly, for VL, conduction velocity increased significantly at 10% (pre: 4.14 (0.22) *vs*. 4.35 (0.19) m s^−1^, *P* = 0.0006, ES = 2.5) and 30% MVC (pre: 4.37 (0.27) *vs*. 4.59 (0.28) m s^−1^, *P* = 0.0004, ES = 2.7) for the matched motor units as well as for the total group of motor units at 10% (pre: 4.17 (0.21) *vs*. post: 4.34 (0.19) m s^−1^, *P* = 0.0008, ES = 2.3) and 30% MVC (pre: 4.39 (0.27) *vs*. post: 4.58 (0.26) m s^−1^, *P* = 0.0018, ES = 2.0).

## Discussion

This study demonstrates the possibility of tracking individual motor units across different days, in humans during voluntary contractions with HDEMG. In Experiment I, without intervention, we were able to effectively track 38.3 % and 40.1 % of the identified motor units across two sessions and 21 (4.9)% and 16.3 (8.9)% across three sessions in the VM and VL, respectively. Moreover, the reliability indexes obtained from tracked motor units were larger than those calculated from the total group of identified motor units and from the unmatched motor units, which strongly confirms a correct tracking. Additionally, the results showed that tracking motor units improved the sensitivity to changes in motor unit conduction velocity following an endurance training intervention, since the changes of conduction velocity of the matched motor units showed a larger effect size compared to the total group of motor units. Taken together, these findings are the first to demonstrate successful tracking of individual motor units recruited during voluntary contractions across several days.

Previous methods have focused on identifying groups of motor units across sessions by using percutaneous electrical stimulation of motor axons (Doherty & Brown 1994, Maathuis *et al*. 2008). This method involves the application of a low‐intensity transcutaneous electrical impulse to the efferent nerve fibres, producing a compound MUAP that can be followed longitudinally. This method has been successfully employed for motor unit number estimation (MUNE) during the progression of neuromuscular disorders, such as amyotrophic lateral sclerosis (Gooch & Harati, 1997). However, this technique does not provide information about central (e.g. discharge behaviour) or peripheral properties (e.g. conduction velocity) of the recorded motor units activated during voluntary contractions (Carroll *et al*. 2011). Thus, the stimulation method is not appropriate for the study of motor unit adjustments during training or other interventions.

The alternative to tracking individual motor units across recordings is to extract a representative sample of motor units and infer population‐behaviour from them (Duchateau *et al*. 2006; Vila‐Cha *et al*. 2010). However, this approach requires a sample large enough to provide reliable information about the properties and behaviour of the motor unit pool (Martinez‐Valdes *et al*. 2016). Moreover, with this approach, a large number of subjects are needed to reach high sensitivity. The method proposedin the current study, conversely, showed the possibility of detecting and monitoring the same motor units across days (up to 2 weeks) with high reliability and sensitivity, which opens new possibilities and opportunities for longitudinal studies.

In comparison to previous single‐channel or intramuscular recordings, HDEMG has the advantage that it provides spatial information as well as time varying aspects of the EMG signal (Blok *et al*. 2002). The likelihood of different motor units having the same spatial action potential representation decreases fast with the number of recording channels (Farina *et al*. 2008). Cescon and Gazzoni (Cescon & Gazzoni, 2010) attempted to track motor units during voluntary contractions using EMG recordings before and after a short‐term bed rest period. The authors analysed motor unit conduction velocity and used a distance measure to discriminate among the different motor units found between trials. However, due to the small number of EMG channels used (7 in the longitudinal direction) and the incomplete decomposition, it was not possible to be certain that matched MUAPs corresponded to the same motor unit, as the authors acknowledged.

In this study, we used a large number of channels in order to exclude the possibility that, due to the volume conductor properties, different motor units showed MUAPs of identical shape (Farina *et al*. 2008). In fact, placing the EMG arrays accurately in the same position for each session and using a relatively large number of channels, it is extremely unlikely that the MUAPs for the decomposition identified in different sessions would show high similarity if they do not correspond to the same motor unit. This property was strongly verified by the reliability and sensitivity analysis which were found both superior for the tracked motor units with respect to the average of all identified motor units, despite the greater sample size of all units. If the motor units were not tracked correctly, the probability of improving reliability and sensitivity of their estimated properties by choosing a smaller subsample of all units would have indeed been negligible. To prove this point further, we also conducted a reliability analysis between random samples of unmatched motor units (the sample size used was similar to the one used for tracked motor units). As expected, the reliability indices decreased even more than those found for the total group of identified motor units, which strongly confirms the accuracy of the tracking.

With our new analysis we were able to identify highly correlated MUAPs for approximately 40–50% of the motor units identified in two sessions and 15–25% of the motor units identified across three sessions, when no intervention was applied. The time‐gap between the different measurement sessions did not influence the number of tracked motor units since the number remained consistent between all two‐sessions comparisons (1–2, 1–3 and 2–3), regardless if they were conducted 1 or 2 weeks apart (See Results and Table 2). This highlights the applicability of the current method for training interventions, since training studies typically last several weeks. However, the number of matched motor units decreased when the procedure was conducted including more than two sessions (e.g. sessions 1–2–3). Finally, we also checked the possibility of tracking motor units across different force levels within a session. Approximately 25% of the motor units identified at each force level (10, 30, 50 and 70% MVC) could be identified at a force level 20% higher (e.g. 10 *vs*. 30% MVC), despite large differences in motor unit recruitment. This shows that the current approach is robust to monitor the properties of the same motor units at different activation levels within a session. Consequently, it is expected that the current approach would still be able to follow motor units when MVC force changes ∼20%.

In terms of reliability, both VM and VL recruitment/de‐recruitment thresholds, mean discharge rates, and conduction velocities showed greater consistency across sessions for the matched motor units compared to the total group of identified motor units. Specifically, ICCs from matched motor units for all variables were substantially greater compared to the ICCs of the total group of identified motor units and unmatched motor units (see Tables 4 and 5), in accordance with the results on SEM (Tables 4 and 5). These observations can be confirmed further by the fact that these reliability indices were as large (or even larger) than the reliability indices obtained from a population of motor units during a sustained isometric contraction (Martinez‐Valdes *et al*. 2016). It is important to note that during ramped contractions, as analysed in this study, motor unit firing behaviour is inherently more variable across the population than during constant‐force isometric contractions (Enoka, 1995). For example, discharge rates of motor units (within a subject) are less correlated during ramped contractions than during constant‐force contractions (Tenan *et al*. 2014). Therefore, the fact that we still found high cross‐session reliability in the present study would be extremely difficult to explain unless matched MUAPs belonged to the same motor units. In fact, there would be no reason for an increase in reliability of measures of motor unit properties when selecting a subset of these units unless they are correctly tracked across sessions, as confirmed by the low reliability levels observed for unmatched motor units.

To show a potential application of the method as well as its sensitivity, we conducted a short‐term high volume endurance training intervention (Experiment II), using a protocol that previously showed an increase of endurance performance, vasti muscle oxidative capacity (Gibala *et al*. 2006) and Na^+^–K^+^ ‐ATPase activity (Green *et al*. 2004), in just 2 weeks. Since changes in oxidative capacity and Na^+^–K^+^ ‐ATPase activity have been suggested as one of the main factors influencing motor unit conduction velocity during submaximal isometric contractions following endurance training (Vila‐Cha *et al*. 2012), it was hypothesized that our protocol would result in an increase in motor unit conduction velocity. Indeed, motor unit conduction velocity increased for both muscles (VM and VL) after the training intervention. However, the magnitude and significance of the detected change differed according to the approach used to assess the motor units. For instance, when matched motor units were used, all the subjects showed a systematic and clear increase in motor unit conduction velocity at 10% and 30% MVC for VM, with high statistical significance and a large effect size (Fig. 4 *A*). However, no statistical difference was observed when using the total group of motor units (Fig. 4 *A* and 4 *B*), with one subject even showing an effect in the opposite direction (Fig. 4 *A*, right). Even though the total group of identified motor unit results for VM were close to reaching statistical significance, it is worth noting that the results for the matched motor units presented an effect size which was almost double than that of the total motor units (matched units ES: 1.8 and 2.4, averaged units ES: 1.2 and 1.1, at 10% and 30% MVC, respectively). Taken together, these results show the impact of the proposed tracking method, which increases the sensitivity to monitor longitudinal changes in motor unit properties. The large number of identified and tracked motor units made available by our technique is critical for obtaining the statistical power needed to support conclusions about motor unit adaptations to training, rehabilitation, or disease (Carroll *et al*. 2011; Button *et al*. 2013; Heroux & Gandevia, 2013).

As representatively shown in the present study, the current method can be applied to the study of motor unit adaptations to training interventions (e.g. resistance or endurance training), but could also be extended to monitor different stages of rehabilitation within the context of injury or disease. For example, the tracking of individual motor unit properties (from low to high threshold motor units) could be of great benefit in characterizing discharge characteristics and muscle‐fibre membrane properties during the progression of neuromuscular disorders (which has not yet been possible with any of the currently available methods). Furthermore, our tracking procedure allows the absolute recruitment threshold force to be measured across sessions without the need to normalize it to %MVC force, providing accurate information about the force capacity of each motor unit. Regarding resistance training, many authors have used surface EMG recordings to attribute early strength gains to neuromuscular adaptations (Folland & Williams, 2007). However, due to the many factors influencing surface EMG amplitude measures (see Farina *et al*. (2004) for review), the evidence is equivocal (Folland & Williams, 2007). Although there are some studies reporting changes in motor unit behaviour following training, demonstrated through intramuscular EMG recordings, the results are not in agreement between studies (Rich & Cafarelli, 2000; Kamen & Knight, 2004; Pucci *et al*. 2006; Vila‐Cha *et al*. 2010), probably due to the small number of motor units that can be identified with this technique and the impossibility of tracking them. Conversely, the current approach could provide clearer evidence of motor unit changes occurring after training interventions since the same motor units can be followed across the intervention. A number of studies have successfully used HDEMG to accurately extract motor unit activity in a number of neuromuscular disorders in single experimental sessions (Holobar *et al*. 2012; Dideriksen *et al*. 2015; Li *et al*. 2015). Our study suggests that these investigations can be extended to include longitudinal characterization of individual motor unit properties in clinical populations.

Some limitations of the proposed approach need to be discussed. In the current study, the motor unit tracking procedure was only applied across sessions that were 2–2.5 weeks apart, during which changes in muscle morphology were not expected. Since changes in muscle morphology (e.g. muscle architecture and cross‐sectional area) influence MUAP shapes, the number of motor units tracked by the algorithm would presumably decrease if the muscle structure changes considerably. However, muscle structural changes, i.e. following resistance exercise (Narici *et al*. 1996; Aagaard *et al*. 2001; McCarthy *et al*. 2002) may not always impact the MUAP shape substantially. As shown in Fig. 3, the present method can successfully track motor units showing large changes in conduction velocity ( > 10%). Moreover, the algorithm can also track motor units between force levels that differ by ∼20% (Table 6). Since motor unit conduction velocity adjustments > 10% and increases in MVC force > 20% are only expected after approximately 6–8 weeks of resistance training (McCarthy *et al*. 2002; Aagaard, 2003; Vila‐Cha *et al*. 2010), it is very likely that the present method can successfully track motor units during longer training interventions than the one shown in this study. A direct evaluation of the method for longer interventions is, however, needed. Similarly, future tests should analyse the possibility of tracking motor units in pathological conditions, such as during the progression of amyotrophic lateral sclerosis (ALS) over long periods of time (van Dijk *et al*. 2010).

The lower number of motor units identified for the vasti muscles with respect to other muscles (e.g. tibialis anterior; Castronovo *et al*. 2015) has been reported previously with a similar blind source separation decomposition method (Watanabe *et al*. 2013; Martinez‐Valdes *et al*. 2016). Differences in muscle fibre architecture across muscles may explain the variability of the identified motor unit sample size across muscles. For example, the tibialis anterior and the gastrocnemius muscles have signal characteristics (Barbero *et al*. 2012) that positively influence the decomposition (less spatially correlated recordings), with respect to muscles such as the vasti or biceps bracchi (Piitulainen *et al*. 2012) that present EMG signals with a higher spatial correlation.

Finally, although occasional, there were a small number of trials (∼15%) where motor units presented multiple matches with a cross‐correlation coefficient > 0.8. As commented above, this could be due to the high spatial correlation that the vasti muscles present. However, the algorithm always selected the highest cross‐correlated source, which prevented the chance of having double matches. The observation of this high correlation between multiple pairs of identified MUAPs indicated the occasional similarity of MUAPs belonging to different motor units. Some degree of similarity is expected and decreases consistently with the number of channels, being negligible for a large number of channels and/or for muscles resulting in low spatial correlation in EMG recordings (Farina *et al*. 2008).

### Conclusion

This study presents and validates, for the first time, a method for processing HDEMG in humans that allows the tracking of the same motor units longitudinally during voluntary contractions performed in different sessions, separated by weeks. This method provides new opportunities to track adaptations of the same motor units over time *in vivo*, as would be required in longitudinal interventions or during the progression of neuromuscular disorders.

## Additional information

### Competing interests

The authors declare that they have no competing interests.

### Author contributions

Experiments were performed at the Institute of Neurorehabilitation Systems, University Medical Center Göttingen, Georg‐August University, Göttingen, Germany and at the Department of Sports Medicine and Sports Orthopaedics, University of Potsdam, Potsdam, Germany. E.M.‐V., F.N., C.M.L, D.Fal, F.M. and D.Far. designed research; E.M.‐V. and C.M.L. performed experiments; E.M.‐V., F.N. and D.Far. analysed and interpreted data; E.M.‐V., F.N., C.M.L., D.Fal and D.Far. contributed to the drafting of the article. All authors have read and approved final submission. All authors agree to be accountable for all aspects of the work, ensuring that questions related to the accuracy or integrity of any part are appropriately investigated and resolved. All persons designated as authors qualify for authorship, and all those who qualify for authorship are listed.

### Funding

This project was funded by the European Research Council Advanced Grant DEMOVE (contract no. 267888) (D. Farina). E. Martinez‐Valdes was supported by a PhD scholarship from the University of Potsdam, based on the postgraduate funding regulations of the federal state of Brandenburg, Germany. F. Negro was funded by the European Union's Horizon 2020 research and innovation programme under the Marie Skłodowska‐Curie grant agreement No 702491 (NeuralCon).

## Appendix A

Multichannel EMG signals can be described mathematically as convolutive mixtures with finite impulse response filters (motor unit action potentials). They can be represented as a linear and an instantaneous mixture of an extended vector of sources (motor unit spike trains) that include the original sources and their *L*–1 delayed versions, where *L* is the length of the filters (Negro *et al*. 2016). This leads to the following extended observation vector for channel *i*:

(A1) $$\begin{array}{c} {\tilde{x}}_{i}(k)=[x_{i}(k)x_{i}(k-1),...,x_{i}\left(k-R\right)],\;i=1,...,m, \end{array}$$

After the extension of the observations, we also have:

(A2) $$\begin{array}{ccc} {\tilde{s}}_{j}\left(k\right) & = & [s_{j}\left(k\right),s_{j}\left(k-1\right),...,s_{j}\left(k-L-R+1\right)],\\ j & = & 1,...,n, \end{array}$$

and

(A3) $$\begin{array}{ccc} {\tilde{n}}_{i}\left(k\right) & = & \left[n_{i}\left(k\right),n_{i}\left(k-1\right),...,n_{i}\left(k|-R\right)\right],\\ i & = & 1,...,m, \end{array}$$

Where *k* is the discrete time, *x_i_* is the EMG signal recorded at channel *i*, *s_j_(k)* the *j*‐th source (motor unit spike train) and *n_i_* the additive noise at channel *i*‐th. Therefore, the extended model becomes:

(A4) $$x\left(k\right)=\left[Hs(k)+n(k)\right]\;k=0,...,D_{R},$$

with

$$s\left(k\right)={\left[s_{1}\left(k\right),s_{2}\left(k\right),...,s_{n}\left(k\right)\right]}^{T},$$

$$x\left(k\right)={\left[x_{1}\left(k\right),x_{2}\left(k\right),...,x_{m}\left(k\right)\right]}^{T},$$

$$n\left(k\right)={\left[n_{1}\left(k\right),n_{2}\left(k\right),...,n_{m}\left(k\right)\right]}^{T},$$

$$\begin{array}{c} h_{ij}=\left[\begin{array}{cccccc} h_{ij}\left[0\right] & \cdots & h_{ij}\left[L-1\right] & 0 & \cdots & 0\\ 0 & \ddots & \ddots & \ddots & \ddots & \vdots \\ \vdots & \ddots & \ddots & \ddots & \ddots & 0\\ 0 & \cdots & 0 & h_{ij}\left[0\right] & \cdots & h_{ij}\left[L-1\right] \end{array}\right], \end{array}$$

$$\underline{\underline{\tilde{H}}}=\left[\begin{array}{ccc} {\tilde{h}}_{11} & \cdots & {\tilde{h}}_{1n}\\ \vdots & \ddots & \vdots \\ {\tilde{h}}_{m1} & \cdots & {\tilde{h}}_{mn} \end{array}\right],$$

where *D_r_* is the duration of the recording and *h_ij_* the action potential of the *j*‐th motor unit recorded at the channel *i*. In order to solve the inverse problem, the number of extended measurements *R* should be higher than the number of sources *n* multiplied by the length of the filters *L* (MUAP shapes).

The instantaneous model described by Eqn (A4) can be inverted to recover the matrix of the extended sources using the fixed point optimization procedure and an appropriate cost function following the spatial whitening procedure (Negro *et al*. 2016). Since the inverse of Eqn (A4) may have a relatively large space of possible solutions, the procedure aims to find the sources ${w_{i}}^{T}z$, where ***z*** are the whitened extended measurements and *w_i_* the projection vector (filter) of the *i*‐th source, that maximize the non‐Gaussianity measure employed by the selected cost function. In this framework, the projection vectors are equivalent to multidimensional filters that extract sparse solutions when applied to the whitened extended measurements. In this study, this method, that we call here ‘full decomposition’, was applied to the first recording session. In the following sessions, we modified the algorithm to identify projection vectors (filters) *w_i_* that would maximize both the non‐Gaussianity of the extracted *i*‐th source and the similarity with the previously identified motor units in the first session. The similarity was estimated by cross‐correlation between the de‐whitened projecting vectors (original multidimensional filters before the whitening procedure or de‐correlation) with a threshold of 0.8. Each time the threshold was crossed, the discharge times of the identified source were removed from the following iterations. The approach is called Sparse Deflation (Natora & Obermayer, 2011) and provides an optimal extraction scheme for sparse signals (e.g. motor unit spike trains) that avoids the convergence to the same solution multiple times. In the tracking application, indeed, the subtraction of the sources in the spike train space resulted more efficient. Among all sources, we selected those with the highest similarity measures.
